# Isolation, structural elucidation, and antioxidant potential of phytomelatonin from tomato (*Solanum lycopersicum* L.) leaves

**DOI:** 10.3389/fphar.2026.1818804

**Published:** 2026-06-24

**Authors:** Kumar Varshini, Theivasigamani Parthasarathi

**Affiliations:** Department of Genetics and Plant Breeding, VIT School of Agricultural Innovations and Advanced Learning (VAIAL), Vellore Institute of Technology, Vellore, Tamil Nadu, India

**Keywords:** antioxidants, bioactive, characterization, isolation, liquid chromatography–tandem mass spectrometry, phytomelatonin

## Abstract

Melatonin (N-acetyl-5-methoxytryptamine) is an indoleamine compound present in both animal and plant kingdoms. Melatonin, a potent antioxidant, is currently produced synthetically, which is often associated with the presence of toxic by-products. This study presents a high-yield alternative by isolating and characterizing pure phytomelatonin (PMT) from underutilized agricultural waste, specifically tomato leaves (*Solanum lycopersicum* L.). By utilizing a multistep extraction and purification process, a yield of 53.12 mg of pure phytomelatonin was achieved. The structural elucidation and purity of the isolate were confirmed through multiple analytical techniques, including ultraviolet–visible (UV–VIS) spectroscopy, Fourier-transform infrared spectroscopy (FT-IR), nuclear magnetic resonance (NMR) spectroscopy, proton nuclear magnetic resonance (^1^H NMR), carbon-13 nuclear magnetic resonance (^13^C NMR), and liquid chromatography–tandem mass spectrometry (LC–MS/MS). The plant-derived phytomelatonin exhibited potent ABTS radical scavenging activity with near equivalence to the synthetic standard while also demonstrating strong DPPH radical scavenging activity with comparatively higher efficiency, maintaining >90% inhibition at elevated concentrations. This research validates the isolation of phytomelatonin from tomato leaves, positioning agricultural by-products as a sustainable and bioavailable source of this valuable bioactive molecule. These findings provide a foundation for future functional and exploration of melatonin’s role in the pharmaceutical, nutraceutical, and agricultural industries.

## Graphical Abstract

**Figure:**
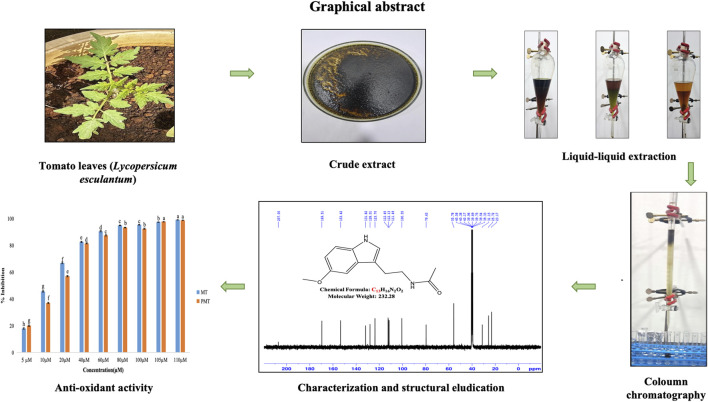
Extraction, characterization, and bioactivity assessment of isolated phytomelatonin from tomato leaves.

## 1 Introduction

Melatonin (N-acetyl-5-methoxytryptamine) is an indoleamine molecule widely distributed in plants, animals, bacteria, and fungi. Primarily, its occurrence was confirmed in the pineal gland of animals, where it is responsible for various metabolic functions, including the regulation of circadian rhythms, seasonal reproduction, immune responses, and sleep–wake cycles (Reiter et al., 1995). The discovery of melatonin in the plant kingdom has established it as a critical botanical regulator. To distinguish its specific roles in plants and its origins from animal-derived melatonin, the term phytomelatonin (PMT) is used to refer specifically to melatonin synthesized by plants (Mannino et al., 2021). Additionally, phytomelatonin plays diverse roles not only in development and growth but also in imparting tolerance to abiotic and biotic stress. Over the past 2 decades, phytomelatonin has emerged as a multifunctional regulatory molecule responsible for stress tolerance, development, and plant growth (Arnao et al., 2007).

Phytomelatonin possesses a strong antioxidant property, thereby efficiently scavenging reactive nitrogen species (RNS) and reactive oxygen species (ROS), including superoxide anions (O_2_^−^), hydroxyl radicals (OH^-−^), hydrogen peroxide (H_2_O_2_), nitric oxide (NO), and peroxynitrite (ONOO^−^), thereby protecting the cellular membranes from oxidative damage (Tan et al., 1999).

Mechanistically, this radical-quenching efficiency is primarily attributed to the high electron-donating capacity of the 5-methoxyindole ring. Unlike many conventional antioxidants, phytomelatonin operates through a continuous scavenging cascade. In this process, a single phytomelatonin molecule can neutralize multiple free radicals because its primary metabolites, formed during the initial scavenging reaction, retain significant antioxidant capacity. This hierarchical defense mechanism ensures that cellular membranes are protected from oxidative damage even at low endogenous concentrations (Khanna et al., 2023; Poeggeler et al., 2002; Kukula-Koch et al., 2021).

Apart from stress conditions, phytomelatonin activity is also enhanced under normal conditions to maintain cellular redox homeostasis through its antioxidant activity (Zhang et al., 2015). Other major functions include the regulation of physiological and developmental processes such as seed dormancy, chlorophyll preservation, root elongation, delayed leaf senescence, stomatal regulation, and fruit ripening (Arnao et al., 2007). Furthermore, phytomelatonin interacts with plant hormones such as ethylene, auxins, abscisic acid, and gibberellins, forming a crucial signaling network that coordinates plant development and stress responses (Zhang et al., 2015).

Phytomelatonin acts synergistically with auxins to promote root organogenesis and lateral root development, mimicking the physiological effects of indole-3-acetic acid (IAA) (Arnao et al., 2019; Pelagio-Flores et al., 2012). Conversely, it often acts antagonistically toward ethylene and abscisic acid (ABA) to delay leaf senescence and mitigate the inhibitory effects of drought stress (Ali et al., 2023). For instance, phytomelatonin interacts with the PMTR1 receptor-mediated signaling pathway to regulate H_2_O_2_ production, which modulates stomatal closure (Wei et al., 2018). Furthermore, it enhances seed germination by maintaining a synergistic balance with gibberellins (GA) while simultaneously downregulating ABA levels through the transcriptional regulation of catabolic genes (Zhang et al., 2014). These responses are fine-tuned by the modulation of specific transcription factors, such as WRKY and MYB, which orchestrate the expression of antioxidant-related genes during stress (Rachappanavar, 2025).

Recent research suggests that phytomelatonin also modulates gene expression by transcription factors and antioxidant-related genes (Wei et al., 2018). These mechanisms make phytomelatonin a natural bioactive compound for agricultural and nutraceutical applications.

In plants, phytomelatonin is synthesized via the tryptophan pathway, progressing through tryptamine, serotonin, N-acetylserotonin before finally forming N-acetyl-5-methoxytryptamine (melatonin). Unlike animals, in which melatonin biosynthesis is largely restricted to the pineal glands, plants synthesize phytomelatonin in various cellular compartments, including the endoplasmic reticulum, cytoplasm, mitochondria, and chloroplasts (Back et al., 2016).

Major sites with the highest concentrations of phytomelatonin are typically the mitochondria and chloroplasts, where ROS production is higher (Tan et al., 2002). The distribution of phytomelatonin varies significantly among plant species, tissues, developmental stages, and environmental conditions, ranging from picograms per gram (pg/g) to micrograms per gram (μg/g) of fresh weight (Arnao et al., 2007). Seeds, young leaves, and reproductive tissues often show elevated levels, reflecting phytomelatonin’s roles in development and stress protection (Tan et al., 1999).

The Solanaceae family is widely recognized as a diverse source of high-value secondary metabolites, such as alkaloids, flavonoids, and potent antioxidants. While numerous crops of this family are known to synthesize melatonin, including peppers (*Capsicum annuum*; 31–93 ng/g dry weight in seeds) (Arnao, M. B., & Hernández-Ruiz, J. 2014), potatoes (*Solanum tuberosum*; 0–3 ng/g of dry weight in tissues) (Badria, 2002; Dubbels et al., 1995), and brinjal (*Solanum melongena*; typically at lower levels), tomato (*Solanum lycopersicum*) contains measurable concentrations of phytomelatonin throughout its tissues, particularly in fruits (7.5–250 ng/g dry weight) and leaves (0.03–142.5 ng/g). However, in general, the concentration of phytomelatonin is higher in leaves than in fruits (Arnao et al., 2007). Among the various horticultural crops cultivated globally, tomato holds a prominent position, with annual production exceeding 180 million tons (FAOSTAT, 2023). However, tomato cultivation also generates substantial amounts of agricultural green waste, such as leaves and stems, which are often discarded or incinerated. This disposal majorly leads to environmental challenges, especially the generation of greenhouse gases and the loss of other important bio-resources (Almeida et al., 2024).

While significant research has focused on the melatonin content of tomato fruits for human nutrition, recent evidence indicates that phytomelatonin is often more concentrated in vegetative tissues, specifically leaves and stems, where it functions as a primary defense against environmental stressors (Cartika et al., 2025). In Solanaceae crops, these tissues serve as the principal metabolic sink for melatonin synthesis, playing a critical role in mitigating oxidative damage induced by UV radiation and temperature fluctuations (Ahmad et al., 2024).

Transitioning from fruit-centric analysis to the valorization of these residues is, therefore, both mechanistically justified and economically vital (Radić et al., 2024). By repurposing high-volume agricultural waste into a low-cost, bio-sustainable source of natural melatonin, this approach aligns with circular bio-economy principles, reducing environmental pollution while providing high-value raw materials for the pharmaceutical and agrochemical industries (Trombino et al., 2021).

Tomato leaves represent metabolically active photosynthetic tissues that constitute a high-yield yet underutilized source, potentially containing higher concentrations than fruits due to their active secondary metabolism. Although melatonin content in tomato fruits has been documented across cultivars and ripening stages, comprehensive studies on leaf-specific isolation, purification, and structural characterization remain scarce (Cartika et al., 2025).

Despite extensive knowledge of the physiological roles of phytomelatonin and its quantification in various plant tissues, several critical gaps remain. First, standardized protocols for the complete isolation and structural elucidation of phytomelatonin from tomato leaves are limited. Furthermore, current literature primarily employs LC–MS/MS for quantification without purification to homogeneity. Second, while the antioxidant properties of synthetic melatonin (SMT) are well-characterized, experimental validation of the bioactivity of isolated and purified plant-derived phytomelatonin remains limited. Third, comparative antioxidant profiling between synthetic standards and plant-isolated fractions has not been systematically conducted for tomato leaves.

Although melatonin is a well-known biomolecule, its isolation from plant materials, particularly agricultural by-products such as tomato leaves, remains largely unexplored. By transforming these underutilized agricultural by-products into a source of high-value bioactive phytomelatonin, this study provides a sustainable alternative to the production of synthetic melatonin. Thus, phytomelatonin has the potential to be used in the nutraceutical, agricultural biostimulant, and pharmaceutical industries while reducing the environmental footprint. Therefore, the present study focuses on specific objectives, which include the extraction, purification, and isolation of phytomelatonin from tomato leaves, to provide structural characterization of isolated phytomelatonin, using ultraviolet–visible (UV–VIS) spectroscopy, liquid chromatography–tandem mass spectrometry (LC–MS/MS), Fourier-transform infrared (FT-IR) spectroscopy, and proton and carbon-13 nuclear magnetic resonance (^1^H and ^13^C NMR) spectroscopy. This study also aims to compare and evaluate the *in vitro* anti-oxidant potential of isolated phytomelatonin against a synthetic melatonin standard using the ABTS radical scavenging assays. By achieving these objectives, this work contributes to a deeper understanding of phytomelatonin’s role as a natural antioxidant and supports its potential utilization as a plant-derived bioactive compound for the sustainable production of natural melatonin in the pharmaceutical, nutraceutical, and agricultural industries.

## 2 Materials and methods

### 2.1 Plant material collection and authentication

Fresh and healthy leaves of (*S. lycopersicum* L. cv. Red Ball) were collected in August 2025 from plants cultivated under controlled conditions in a shade net house maintained at VIT University, Vellore, India. The geographical coordinates of the collection site were (12.968460762651837° N) latitude and (79.15950067818771° E) longitude. The taxonomic identity of the plant sample was authenticated by Mr Vishnu Walsan K, Research Officer (Botany), at the Central Siddha Clinical Research Institute (CSCARI), Tamil Nadu, India. The voucher specimen (No. 2506012) was prepared, labeled, and deposited in the institutional herbarium for future reference and verification.

### 2.2 Extraction and liquid–liquid fractionation of plant material

#### 2.2.1 Preparation of crude ethanolic extract

The shade-dried and coarsely powdered plant material (100 g) was subjected to extraction by maceration with 90% ethanol in a clean glass container. The solvent mixture was kept undisturbed for 72 h at room temperature with intermittent stirring to attain complete extraction of bioactive constituents, following standard phytochemical extraction procedures (Harborne et al., 1998; Handa et al., 2008). Subsequently, the extract was filtered through muslin cloth followed by Whatman No. 1 filter paper to eliminate particulate matter. The residual plant material was subjected to repeated re-maceration and solvent extraction under identical conditions to maximize extraction efficiency (Azwanida, 2015). The filtrates obtained from each batch were collected, combined, and concentrated under reduced pressure using a rotary evaporator at 40 °C to remove the solvent, yielding a semisolid crude extract. The dried extract was weighed and stored in a sterile container at 4 °C for further phytochemical analysis and bioactivity studies.

#### 2.2.2 Determination of the extraction yield

Extraction yield was calculated using Equation 1:

Y=W1/W2 X100, (1)

where Y represents the percentage extraction yield, W1 is the weight of the crude extract obtained after extraction, and W2 is the weight of the plant material used. The yield calculation followed standard procedures (Pandey et al., 2014).

#### 2.2.3 Liquid–liquid fractionation

Liquid–liquid extraction was carried out to separate the target organic compound based on polarity differences. A portion (5 g) of the crude extract was dissolved in (50 mL) of deionized water and partitioned with solvents of increasing polarity. Initially, a non-polar solvent such as hexane (50 mL) was utilized to separate oils and steroids from the crude extract. Subsequently, chloroform (50 mL) was added to the aqueous phase of the sample for the separation of alkaloids. Finally, the polyphenol was separated by adding an immiscible aqueous solvent such as n-butanol (50 mL). This solvent partitioning strategy was performed according to established natural product isolation principles (Harbone et al., 1998; Sarker et al., 2006; Stalika et al., 2007).

#### 2.2.4 Estimation of melatonin content

Melatonin content in the extracts was estimated by a ninhydrin–sulfuric acid colorimetric method (Amin et al., 2000) with minor modifications. This assay was utilized as a rapid screening tool to evaluate the cumulative concentration of molecules possessing the indole moiety across various solvent systems. Standard solutions of melatonin (25–1,000 μg/mL) were prepared in methanol. An aliquot (500 µL) of standard or sample extract was added to 200 µL of 2% ninhydrin solution (0.2 g in 10 mL ethanol), followed by 300 µL of 96% H_2_SO_4_, vortexed, and incubated for 20 min at room temperature. Optical absorbance was measured at 530 nm using a UV–VIS spectrophotometer, and a calibration curve (A_530_ vs. concentration) was constructed. The phytomelatonin content was expressed as melatonin equivalents (ME) per dry weight (mg ME/g dry weight) of plant material. This approach allowed for the quantitative assessment of the total indole-rich fraction, including melatonin and related indole-based metabolites, prior to specific structural characterization.

#### 2.2.5 Thin-layer chromatography analysis

The presence of alkaloids, including melatonin, in the sample was evaluated using thin-layer chromatography (TLC) following standard phytochemical procedures (Sarker et al., 2012; Sasidharan et al., 2011).

A melatonin standard solution (1 mg/mL in methanol) and test samples (1 mg/mL) comprising crude extract in hexane, chloroform, methanol, and aqueous fractions were applied onto 10 × 5 cm silica gel GF254 plates using a 5 µL Hamilton syringe with a Linomat V automated spray-on applicator. Each sample (5 µL) was applied as a 5 mm band, maintaining a 5 mm distance between band centers.

The elution was carried out for approximately 20 min in a pre-saturated vertical glass chamber containing a mobile phase consisting of toluene: ethyl acetate: formic acid (5:4.5:0.5, v/v/v), as described in standard TLC protocols for indole alkaloids (Wagner et al., 2009). The solvent front migration distance was recorded. After elution, the plates were air-dried and visualized under white light and long-wavelength ultraviolet light (366 nm) using a TLC visualizer. TLC experiments were performed in triplicate to ensure reproducibility of the separation.

The retention factor (Rf) values were calculated using Equation 2:

$$\text{Rf}=\frac{\text{Distance}\;\text{moved}\;\text{by}\;\text{the}\;\text{sample}}{\text{Distance}\;\text{moved}\;\text{by}\;\text{the}\;\text{solvent}\;\text{front}}.$$ (2)

Rf values of sample bands were compared with that of the melatonin standard for identification purposes (Ansari et al., 2010).

#### 2.2.6 Isolation of melatonin using column chromatography

The fraction with the highest melatonin was subjected to silica gel column chromatography for the isolation of melatonin, following standard natural product separation protocols (Sarker et al., 2012).

Silica gel (60–120 mesh) was used as the stationary phase, and the column was packed by the wet packing method using chloroform as the initial solvent. The sample was pre-adsorbed onto a small quantity of silica gel and carefully loaded onto the column.

Elution was carried out using a gradient solvent system of increasing polarity. The column was first eluted with chloroform–ethyl acetate mixtures (0:100 → 100:0, v/v), followed by methanol–water mixtures (100:0 → 80:20, v/v) to facilitate the separation of moderately polar indoleamine compounds such as melatonin. A total of sixteen fractions (5 mL each) were collected and monitored at a flow rate of approximately 1 mL/min.

The collected fractions were assessed by TLC on silica gel GF254 plates using toluene: ethyl acetate: formic acid (5:4.5:0.5, v/v/v) as the mobile phase (Table 1). The melatonin standard was used for comparison. Developed plates were visualized under UV light at 366 nm, and fractions exhibiting similar fluorescence characteristics and Rf values corresponding to the standard were identified.

The fractions that matched the standard melatonin in terms of fluorescence and Rf value were pooled and subjected to further purification. The pooled fractions were re-analyzed by TLC to confirm homogeneity and subsequently crystallized from methanol by slow evaporation at room temperature. The isolated phytomelatonin was finally confirmed by spectrophotometric characterization.

**TABLE 1:** Composition of the mobile phase used for TLC analysis of melatonin.

Sample code	Sample name	Solvent system	Rf (UV 254 nm)
A	Standard (melatonin)	Toluene: ethyl acetate: formic acid (5:4.5:0.5)	0.29 ± 0.01
B	Crude extract		0.30 ± 0.01
C	Hexane fraction		-
D	Chloroform fraction		0.30 ± 0.01
E	n-Butanol fraction		-
F	Aqueous fraction		-

#### 2.2.7 Spectroscopic characterization

The absorbance of the isolated compound (in methanol solvent) was recorded using a Shimadzu UV-1280 spectrophotometer (wavelength range 190–1,100 nm) over the range of 200–400 nm, and λ_max_ values were used to compare the crude extract, fractions, and isolated phytomelatonin. FT-IR spectra were obtained using a Shimadzu IRAffinity-1 spectrophotometer in the range of 4,000–400 cm^-1^, and characteristic bands were assigned to confirm functional groups.

The NMR spectra (^1^H and ^13^C) of the isolated compound were recorded using a Bruker Avance III 400 MHz. Chemical shifts (δ) are reported in ppm, and coupling constants (J) are reported in Hz. LC-MS/MS analysis was performed on a Shimadzu −8045 RX quadrupole coupled mass spectrometer equipped with an ESI source operating in positive ion mode. Separation was achieved on a C_18_ column (2.1 × 100 mm, 1.8 µm) using a water (0.1% formic acid) acetonitrile gradient at a flow rate of 0.3 mL/min, and melatonin was quantified by multiple reaction monitoring (MRM) using an external calibration curve.

#### 2.2.8 Evaluation of *in-vitro* antioxidant activity

With a few minor adjustments, the ABTS radical cation decolorization assay was used to assess the antioxidant activity of the isolated phytomelatonin (Pérez et al., 2020). ABTS^+^ was produced by mixing 7 mM ABTS with 2.45 mM K_2_S_2_O_4_ (1:1, v/v) and allowing the mixture stand at room temperature for 12–16 h in the dark. The ABTS^+^ solution was diluted with methanol until its absorbance at 734 nm was 0.70 ± 0.02. By adding 0.1 mL of sample or melatonin standard (10–100 μg/mL) to 3.9 mL of ABTS^+^ solution, the mixture was kept at room temperature for 6 minutes. A UV–VIS spectrophotometer was used to measure absorbance at 734 nm.

The free radical scavenging activity of purified phytomelatonin and synthetic melatonin was determined using the 2,2-diphenyl-1-picrylhydrazyl (DPPH) assay, as described by Brand-Williams et al. (1995) with minor modifications. In brief, a 0.1 mM DPPH solution was prepared in methanol. Various concentrations of the purified isolate and synthetic melatonin (5 μm–110 μm) were mixed with 2 mL of the DPPH solution. The mixture was shaken vigorously and incubated in the dark at room temperature for 30 min. The reduction of the DPPH radical was monitored by measuring the absorbance at 517 nm using a UV–VIS spectrophotometer. All measurements were taken in triplicate (n = 3).

## 3 Statistical analysis

Statistical analysis was carried out using ANOVA, followed by Tukey’s *post hoc* test, in JMP Pro software. A *p*-value of < 0.05 is considered statistically significant.

## 4 Results

### 4.1 Extraction yield

Air-dried powdered leaf material (100 g) was extracted with 90% ethanol by maceration. The extract was filtered and concentrated under reduced pressure using a rotary evaporator at 40 °C to prevent thermal degradation of heat-sensitive constituents, yielding a semisolid crude extract. The mass of the crude extract obtained was 16 g, corresponding to an extraction yield of 16.05% (w/w) relative to the initial dry weight of the plant material.

### 4.2 Estimation of melatonin

**FIGURE 1:**
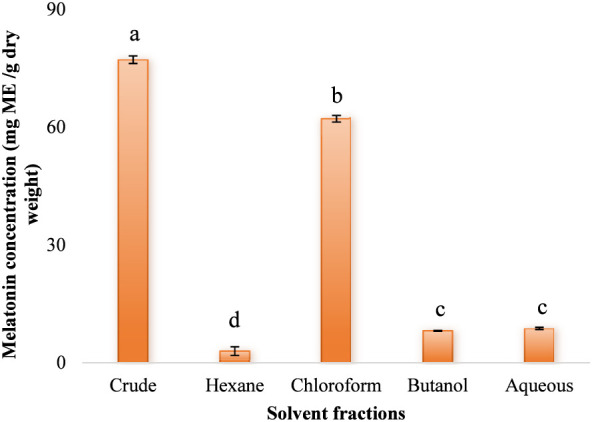
Melatonin concentration (mg ME/g dry weight) of crude tomato leaf extract and its solvent fractions, determined by the ninhydrin–sulfuric acid colorimetric method. Values are presented as the mean ± SD (n = 3). Different letters indicate significant differences between fractions (one-way ANOVA, followed by Tukey’s test, *p* < 0.05).

The melatonin content was estimated in various solvent fractions. Initially, the crude tomato leaf extract contained (77.16 ± 1.68 mg ME/g dry weight) of melatonin equivalents (ME) per gram of dry weight, which was determined by the ninhydrin–sulfuric acid colorimetric method at 530 nm (Figure 1). The solvent fractionations demonstrated that the concentration of melatonin equivalents (ME) per gram of dry weight was high in the chloroform fraction (62.29 ± 1.43 mg ME/g dry weight), whereas the butanol and aqueous fractions exhibited an average level (8.14 ± 0.16 mg ME/g dry weight and 8.75 ± 0.49 mg ME/g dry weight, respectively). In addition, the hexane fraction contained a very low melatonin content (3.04 ± 1.89 mg ME/g dry weight). These results indicate that melatonin is soluble in moderately polar solvents under fractionation conditions (Yeligar et al., 2017). These results, which reflect the cumulative abundance of indole-based metabolites, rather than melatonin specifically, served as a quantitative guide for the subsequent specific isolation and characterization of phytomelatonin.

### 4.3 Thin-layer chromatography analysis

**FIGURE 2:**
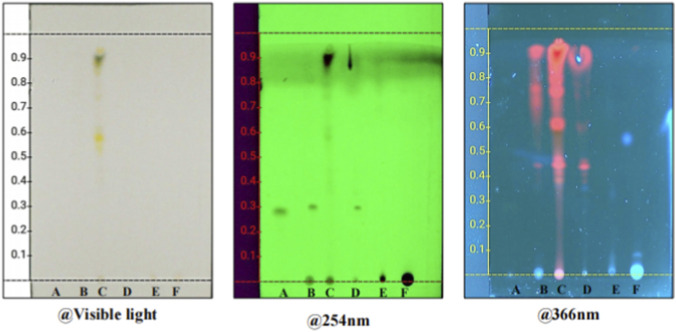
Thin-layer chromatography analysis of tomato leaf extracts and fractions for phytomelatonin detection. Lanes: (A) melatonin standard, (B) crude ethanolic extract, (C) hexane fraction, (D) chloroform fraction, (E) butanol fraction, and (F) aqueous fraction.

Under UV visualization at 254 nm (Figure 2), the melatonin standard (Lane A) (Rf value = 0.29) ± 0.01 and the corresponding bands in the crude extract (Lane B) and chloroform fraction (Lane D) (Rf value = 0.30 ± 0.01) appeared as dark quenching spots against the green fluorescent silica matrix. This quenching behavior is characteristic of the indole ring’s UV absorption at short wavelengths, which interferes with the plate’s background fluorescence.

Under 366 nm UV illumination, bands at the corresponding Rf values were observed in the crude extract and chloroform fraction, while no clear bands were detected in the hexane fraction (Lane C). The butanol (Lane E) and aqueous (Lane F) fractions did not show clearly resolved bands at the corresponding Rf value. These dual-wavelength observations provide preliminary confirmation of indole-based bioactives in the enriched fractions.

### 4.4 Isolation of melatonin using column chromatography

The chloroform fraction 6.8 g, obtained from 16 g crude extract derived from 100 g dried tomato leaves, was subjected to silica gel column chromatography using dual-gradient elution: chloroform: ethyl acetate (0:100 → 100:0), followed by methanol: water (100:0 → 80:20). A total of 16 fractions (5 mL each) were collected at a flow rate of approximately 1 mL/min and screened via TLC using toluene: ethyl acetate: formic acid (5:4.5:0.5, v/v/v) alongside a melatonin standard (Table 1). Fractions 3 and 4 exhibited fluorescence characteristics and Rf values comparable to the standard melatonin and were, therefore, pooled.

The pooled fractions were further purified by preparative TLC (10 lanes) using the same solvent system (Supplementary Figure S1). The corresponding bands were scraped, eluted, and crystallized by slow evaporation of methanol at room temperature. This process yielded 53.12 mg of purified melatonin, corresponding to 0.777% (w/w) of the chloroform fraction and 0.05312% (w/w) relative to the initial dried plant material.

## 5 Spectroscopic characterization

### 5.1 UV–visual spectroscopy analysis

**FIGURE 3:**
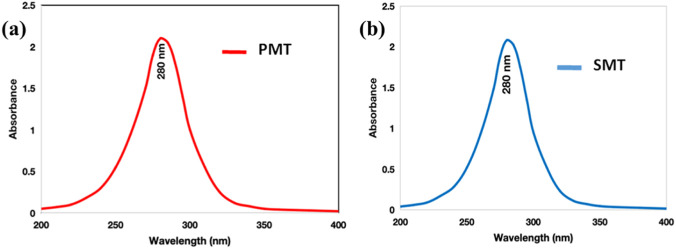
UV–VIS absorption spectrum of **(a)** purified phytomelatonin (PMT) and **(b)** synthetic melatonin (SMT), showing a characteristic absorption maximum (λmax) at 280 nm for both samples.

The UV–VIS absorption spectrum of purified phytomelatonin (PMT) and synthetic melatonin are shown in Figures 3a,b. The spectrum exhibited an intense absorbance in the UV region, producing a single broad peak with a λ_max_ value of 280 nm for both samples. This can be attributed to the π → π∗ transition within its aromatic indole ring system (Singh et al., 2014). Furthermore, previously published studies on melatonin and related indolamines have reported strong absorption peaks at 270 nm and 285 nm (Omer et al., 2021). The observed absorption maximum at 280 nm is, therefore, consistent with published UV–VIS data for melatonin standards and phytomelatonin-rich plant extracts, supporting the qualitative identification of melatonin in the purified sample. This identification was further confirmed by LC–MS/MS and NMR analysis.

### 5.2 FT-IR analysis

**FIGURE 4:**
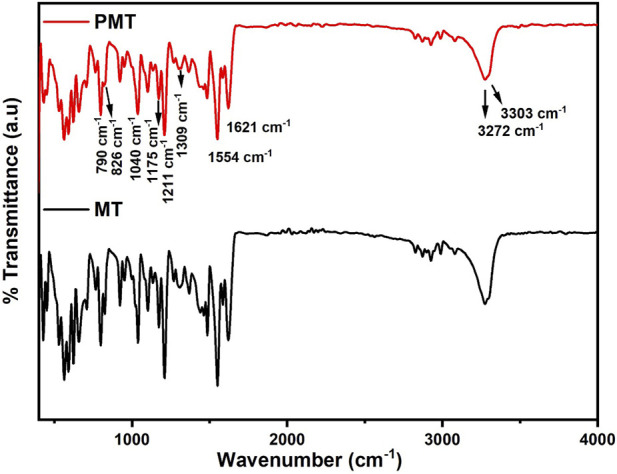
FT-IR spectra of purified phytomelatonin (red) and standard melatonin (black).

The FT-IR spectra of purified phytomelatonin and synthetic melatonin exhibited a high degree of similarity, confirming the presence of identical functional groups and structural features **(** Figures 4)**.** Notably, both the spectra exhibited characteristic absorption bands at 3,300 cm^-1^ (broad N–H stretching vibration of the amide group), 1,650–1,620 cm^-1^ (amide C=O stretching), 1,560 cm^-1^ (aromatic C=C stretching of the indole ring), 1,260 cm^-1^ (asymmetric C–O–C stretching of the methoxy group), and 1,030 cm^-1^ (symmetric C–O–C stretching of the methoxy group) (Singh et al., 2014). These peaks are consistent with the reported FT-IR signatures of melatonin. Collectively, the FT-IR data confirm the successful isolation of phytomelatonin.

### 5.3 LC–MS/MS analysis

**FIGURE 5:**
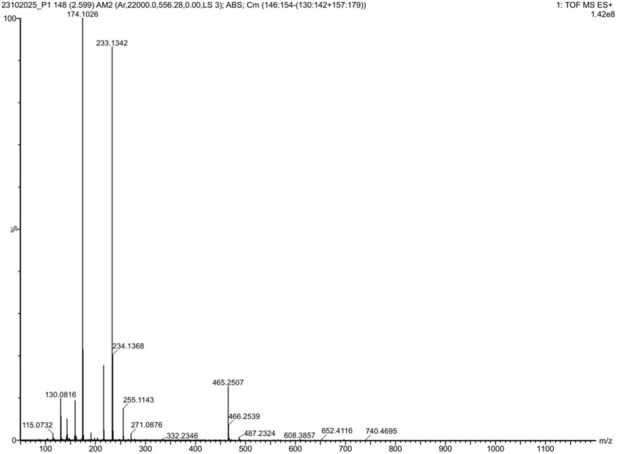
LC–MS chromatogram of purified PMT, showing the target peak at retention time Rt with [M + H]^+^ at m/z 233.1, matching the molecular ion of melatonin.

The mass spectrum of the purified phytomelatonin fraction from positive ESI mode resulted in a molecular ion peak at m/z = 233.13, corresponding to the protonated molecular ion [M + H]^+^ of melatonin (theoretical m/z 233.1). The common fragment ion at m/z 174.10 was also observed (Figure 5) (Pérez et al., 2020).

### 4.4 NMR analysis

**FIGURE 6:**
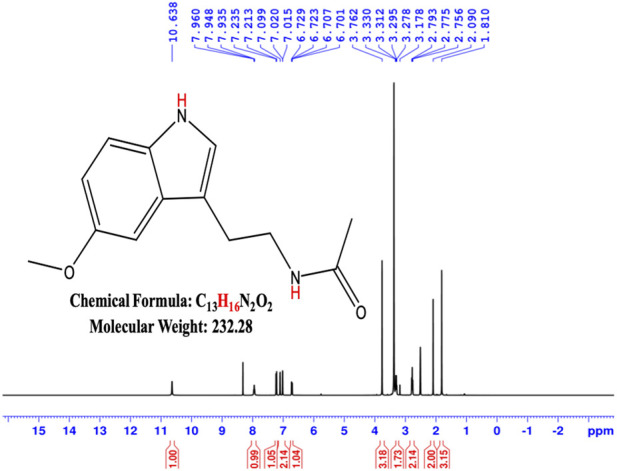
^1^H NMR spectrum of purified PMT in DMSO-d_6_ at 400 MHz.

The structural elucidation of the isolated phytomelatonin was carried out using NMR spectroscopy. In brief, ^1^H NMR and ^13^C NMR were carried out to investigate the chemical environment of the isolated sample. Key signals are assigned to characteristic protons of the N-acetyl-5-methoxytryptamine structure. The ^1^H NMR spectrum of the isolated compound from tomato leaves in DMSO-d_6_ (400 MHz) further confirmed that the isolated molecule is chemically identical to melatonin (Figure 6). The spectrum displayed a singlet at δ ≈ 1.8 ppm (3H) that can be assigned to the N-acetyl methyl group and a singlet at δ ≈ 3.7 ppm (3H) corresponding to the methoxy (O-CH 33) protons, together with two sets of methylene protons of the ethylamine side chain resonating between δ ≈ 2.0 and 2.8 ppm. Aromatic protons of the indole ring appeared as multiplet/doublet signals between δ ≈ 6.7 and 7.3 ppm, while a downfield singlet at δ ≈ 7.9 ppm and a broad signal at δ ≈ 10.6 ppm were attributed to the indole and amide NH protons, respectively (Table 2), consistent with literature values of melatonin (Bhattacharjee et al., 2014). Furthermore, the assigned peaks along with the spin multiplicity, integration, and interpretation for ^1^H NMR in Table 2 with reported literature for comparison.

**TABLE 2:** ^1^H NMR data of purified phytomelatonin (N-acetyl-5-methoxytryptamine) in DMSO-d_6_ (400 MHz), compared with existing literature data.

Assignment/Position	Experimental data ^1^H NMR δ (ppm), multiplicity, integration	Literature data ^1^H NMR δ (ppm) (Darwish and Attia, 2012)
Indole-NH	10.41 (s, 1H)	10.62
Amide-NH	8.01 (s, 1H)	7.90
Aromatic H/C	6.70–7.70 (m, 4H)	6.70–7.21
Methoxy	3.79 (s, 3H)	3.72
Aliphatic	3.27 (m, 2H)	3.28
Aliphatic	2.80 (t, 2H)	2.67
Acetyl methyl	1.90 (s, 3H)	1.87

s, singlet; d, doublet; t, triplet.

**FIGURE 7:**
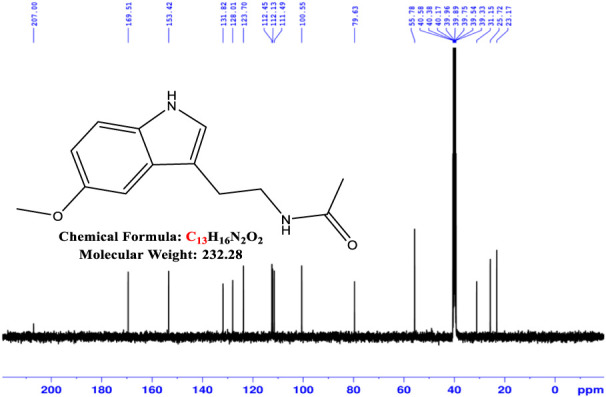
^13^C NMR spectrum of purified PMT in CDCl_3_ at 400 MHz.

Additionally, the ^13^C NMR spectrum of the purified phytomelatonin (PMT, 100 MHz, DMSO-d_6_) showed the expected number of carbon signals for melatonin (C_13_H_16_N_2_O_2_), including resonances for the indole aromatic carbons, the methoxy carbon, the two side-chain methylene carbons, and the N-acetyl carbonyl carbon. The chemical shifts of all carbons were similar to the reported ^13^C NMR data for melatonin, indicating the presence of an indole ring, a methoxy group, and an N-acetyl amide group in the isolated phytomelatonin. The obtained data are consistent with the reported ^13^C NMR data for melatonin (Bhattacharjee et al., 2014), confirming the presence of an indole ring, a methoxy group, and an N-acetyl amide (Figure 7). Furthermore, the assigned peaks and their corresponding ^13^C NMR interpretations are presented in Table 3, alongside reported literature values for comparison.

**TABLE 3:** ^13^CNMR data of purified phytomelatonin (N_acetyl_5_methoxytryptamine) in DMSO_d_6_ (100MHz), compared with the exisiting literature data.

Assignment/Position	Experimental data ^13^C NMR δ (ppm)	Literature data ^13^C NMR δ (ppm) (Văn Thoại Nam, 2013)
Carbonyl	169.51	169.4
C-5	153.42	153.3
C-7a	131.82	131.7
C-3a	128.01	128.1
Aromatic H/C	102.50–123.70	102.3–123.6
Methoxy	55.78	55.7
Aliphatic	39.31	39.4
Aliphatic	25.21	25.4
Acetyl methyl	23.17	23.1

## 6 Evaluation of *in vitro* antioxidant capacity using the ABTS and DPPH assays

The antioxidant capacity of SMT and PMT was evaluated using both ABTS and DPPH radical scavenging assays. In both experiments, activity increased in a concentration-dependent manner from 5 to 110 μM.

**FIGURE 8:**
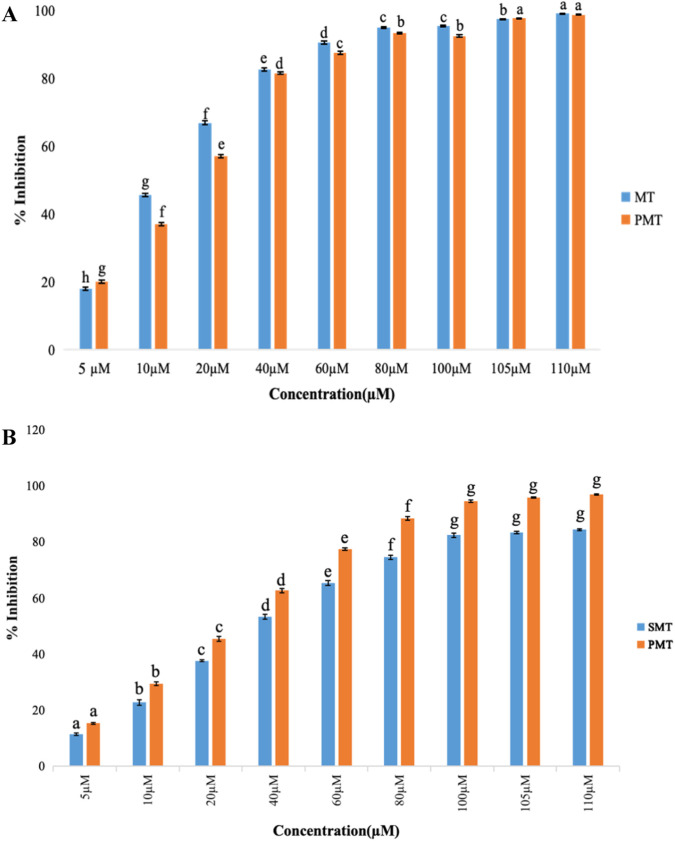
**(A)** ABTS ^+^ radical scavenging activity of standard melatonin (SMT, blue) and purified phytomelatonin (PMT, orange) at concentrations ranging from 5 to 110 μM. Values are presented as the mean ± SE (n = 3). **(B)** DPPH scavenging activity of standard melatonin (SMT, blue) and purified phytomelatonin (PMT, orange) at concentrations ranging from 5 to 110 μM. Values are presented as the mean ± SE (n = 3).

In the ABTS assay (Figure 8A), inhibition reached >90% at concentrations above 60 μM for both compounds, with observed IC_50_ values of 12 μM for SMT and 16 μM for PMT. While SMT showed slightly higher potency in the ABTS environment, the DPPH assay (Figure 8B) revealed a superior performance by the plant-derived variant. Specifically, PMT achieved >90% DPPH inhibition at 80 μM, whereas SMT required 100 μM to achieve the same threshold. The IC_50_ values for DPPH were found to be 25.32 μM for PMT and 34.50 μM for SMT, indicating that PMT is more potent in scavenging DPPH radicals.

Generally, lower IC_50_ values represent higher radical-quenching efficiency (Tan et al., 2003). From these combined experimental results, it can be concluded that isolated phytomelatonin possesses significant antioxidant activity that is not only comparable to but, in certain radical environments, superior to synthetic melatonin. Therefore, these findings emphasize that plant-derived phytomelatonin functions as a highly effective antioxidant, consistent with previous reports highlighting the robust radical scavenging properties of the melatonin molecule (Pérez et al., 2020).

## 7 Discussion

The current extraction and isolation of the phytomelatonin from the tomato plant yielded 53.12 mg of the pure compound. The yield obtained in this study appears comparatively higher; however, variations may arise due to differences in the plant source, extraction methodology, and quantification approaches. For example, the isolation of melatonin from *Crataeva nurvala* yielded 20 mg of pure melatonin (Bhattacharjee et al., 2014). The current study on phytomelatonin isolation demonstrated a 2.6 fold increase, highlighting the potential of tomato-based, high-yield, and cost-effective extraction methods for the production of functional phytomelatonin.

The isolation of melatonin from plant sources is a complex, multi-step approach due to the compound’s low endogenous concentrations and its susceptibility to photo-oxidation. Unlike simple extraction, isolation requires systematic removal of plant interferents such as chlorophyll, carotenoids, and polyphenols (Ye et al., 2017). This is evidenced by the LC–MS/MS data, which provide precise identification and confirm the structural integrity and purity of the isolate by separating contaminants and analyzing the molecular mass. The protonated molecular ion [M + H]^+^ at m/z 233.13 corresponds to the expected molecular mass of 233.1, indicating a high degree of purity and the absence of detectable Na^+^ or K^+^ ions. The fragment ion at m/z 174.10 is significant; this signal corresponds to the loss of the acetamide side chain (CH_3_CONH_2_), leaving the 5-methoxy-indole-methylene cation. This fragmentation pattern is a widely accepted fingerprint in the plant melatonin literature and is used to distinguish melatonin from isomers such as 6-hydroxymelatonin (Ye et al., 2017). Thus, it effectively confirms the isolation of melatonin by distinguishing it from complex plant constituents and potential interferents such as chlorophyll and carotenoids that may complicate its identification in plant tissues.

Moreover, the observed λ_max_ value of 280 nm falls within the characteristic range of indole chromophores, which typically exhibit strong UV absorption at approximately 278–282 nm (Girgin et al., 2016). The FT-IR analysis further provides evidence regarding the functional groups present in the isolated compound. The broad absorption at 3,300 cm^-1^ represents the presence of N-H stretching of the secondary amide, while the sharp peak at 1,630 cm^-1^ represents the amide band stretching. The close agreement of these absorption maxima with those of the synthetic standard confirms that the isolation process preserved the structural integrity of the melatonin molecule, including its acetyl group. Furthermore, the presence of the 5-methoxy group, a crucial structural feature for melatonin’s biological and antioxidant activity, is confirmed by the asymmetric and symmetric C-O-C stretching vibrations at 1,260 and 1,030 cm^-1^, respectively (Bhattacharjee et al., 2014).

The NMR spectroscopic profile (^1^H and ^13^C) reveals a detailed map of the hydrogen and carbon environments, confirming the indole-based structure. The ^1^H NMR singlet at δ ≈ 1.8 ppm corresponds to the protons of the N-acetyl methyl group, while the singlet at δ ≈ 3.7 ppm confirms the methoxy protons. The shift of the indole NH at δ ≈ 10.6 ppm and amide NH at δ ≈ 7.9 ppm represents the characteristics of the melatonin molecule in DMSO-d_6_. These chemical shifts are consistent with those reported in early isolation studies. The ^13^C NMR data provide structural proof, with the carbonyl carbon resonance (amide C=O) appearing in the downfield area, distinct from the aromatic indole carbons (Bhattacharjee et al., 2014).

Biological functional validation of the isolate was achieved through a dual-assay approach involving ABTS and DPPH radical scavenging. Both the phytomelatonin and synthetic melatonin standard demonstrated linear, dose-dependent inhibition across both systems. In the ABTS assay, inhibition exceeded 90%, with synthetic melatonin showing slightly higher potency (IC_50_ = 12 µM) compared to the purified isolate (IC_50_ = 16 µM). Similarly, the DPPH assay confirmed strong scavenging activity, with IC_50_ values of 25.32 μM for phytomelatonin and 34.50 μM for synthetic melatonin. Both values fall within the range of highly potent antioxidants. This sustained potency is attributed to the high electron-donating capacity of the 5-methoxyindole ring and its unique ability to engage in a continuous scavenging cascade (Mohammed et al., 2023). Unlike conventional antioxidants, this mechanism allows a single melatonin molecule to neutralize multiple free radicals by generating stable secondary and tertiary metabolites that remain antioxidative.

Despite melatonin’s known susceptibility to photo-oxidation, the isolate retained over 90% of its antioxidant capacity. This suggests that trace, co-extracted natural antioxidants within the tomato leaf matrix may have provided a protective, synergistic effect during processing (Messina et al., 2001). This occurrence can be further investigated as it could have crucial applications for the industrial shelf-life of plant-derived antioxidants (Arnao and Ruiz et al., 2018). Nevertheless, the results confirm that tomato leaf-derived phytomelatonin retains the full radical-quenching efficiency reported in modern botanical antioxidant studies (Arnao and Hernández-Ruiz, 2014; Ruiz et al., 2016).

A major strength of this study is the shift from simple quantification to homogeneous isolation and structural elucidation through (UV–VIS, FT-IR, MS, and NMR). In addition, the use of agricultural green waste promotes a circular bio-economy, providing a sustainable alternative to the chemical synthesis of melatonin. However, a limitation of this research is the use of a single tomato cultivar. Since endogenous melatonin levels vary based on circadian rhythms and, environmental stressors (Cartika et al., 2025), the total yield may vary depending on the duration of plant growth or geographical location. In conclusion, these findings suggest that tomato leaf by-product is a functional source of phytomelatonin. This work proposes a standardized framework for the pharmaceutical industry to adopt bio-based, natural alternatives, reducing the environmental impact of traditional synthetic production.

## 8 Conclusion

This study successfully isolated and comprehensively characterized phytomelatonin from tomato (*Solanum lycopersicum* L.) leaves using a scalable protocol, yielding 53.12 mg of the pure compound from chloroform fractionation of the crude ethanolic extract. Spectroscopic techniques such as UV–VIS spectroscopy (λ_max_ = 280 nm), FT-IR (characteristic amide NH 3,300 cm^-1^, C=O 1,650 cm^-1^, and indole 1,560 cm^-1^), NMR (^1^H/^13^C matching N-acetyl-5-methoxytryptamine shifts), and LC–MS/MS ([M + H]^+^ m/z 233.13) confirmed the molecular identity. Purified phytomelatonin demonstrated potent antioxidant activity (ABTS IC_50 =_16 μM), comparable to synthetic melatonin standards (IC_50 =_12 μM), with both achieving >90% radical scavenging above 60 μM. Meanwhile, the DPPH assay confirmed strong scavenging activity with IC_50_ values of 25.32 μM) for phytomelatonin and 34.50 μM for synthetic melatonin.

These findings validate tomato agricultural by-products as scalable natural antioxidant platforms for biostimulants (stress tolerance), functional foods (dietary supplements), and pharmaceutical precursors. The established protocols provide a foundational methodology for phytomelatonin research across Solanaceae crops, enabling future physiological, agronomic and nutraceutical applications.

## Data Availability

The datasets presented in this study can be found in online repositories. The names of the repository/repositories and accession number(s) can be found in the article/Supplementary Material.
