# Supplementary figures and images for: Isolation, structural elucidation, and antioxidant potential of phytomelatonin from tomato (Solanum lycopersicum L.) leaves
Source: Front Pharmacol. 2026 Jun 24;17:1818804. doi: 10.3389/fphar.2026.1818804 (PMC13342173; doi:10.3389/fphar.2026.1818804)

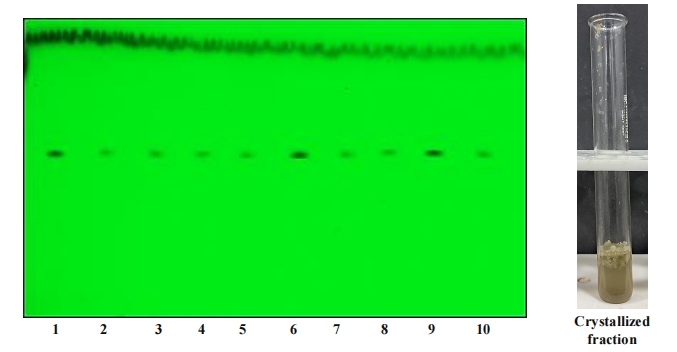

Supplement: Supplementary file 1 [file Image1.tif]
