# Supplementary material for: Isolation, structural elucidation, and antioxidant potential of phytomelatonin from tomato (Solanum lycopersicum L.) leaves
Source: Front Pharmacol. 2026 Jun 24;17:1818804. doi: 10.3389/fphar.2026.1818804 (PMC13342173; doi:10.3389/fphar.2026.1818804)
Supplement: Supplementary file 2 [file DataSheet1.docx]

**Isolation, Structural elucidation and antioxidant potential of Phytomelatonin from Tomato (Solanum lycopersicum L.) Leaves**

**Authors and affiliations**

Kumar Varshini, Theivasigamani Parthasarathi

Department of Genetics and Plant Breeding, VIT School of Agricultural Innovations and Advanced Learning (VAIAL), Vellore Institute of Technology, Vellore, Tamil Nadu, India.

**Correspondence**

Theivasigamani Parthasarathi*
parthasarathi.t@vit.ac.in

**Supplementary -1**


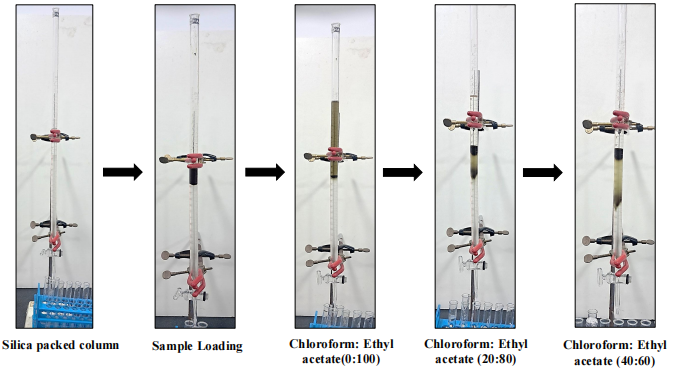


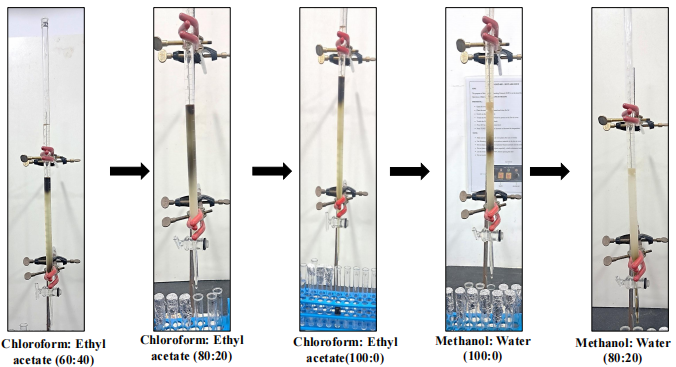


The chloroform fraction subjected to silica gel column chromatography using dual-gradient elution: Chloroform: Ethyl acetate (0:100 → 100:0), followed by Methanol: Water (100:0 → 80:20).


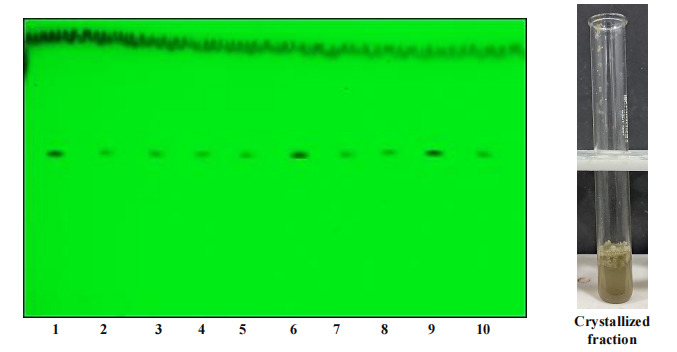


Preparative TLC plate (10 lanes) used for further purification of pooled fractions 3 and 4 from column chromatography, visualized under UV light at 366 nm. Mobile phase: toluene: ethyl acetate: formic acid (5:4.5:0.5, v/v/v).
